# RE: Extracorporeal circulatory systems in the interhospital transfer of critically ill patients: experience of a single institution

**DOI:** 10.4103/0256-4947.60529

**Published:** 2010

**Authors:** Peter Feindt, Hannan Dalyanoglu, Artur Lichtenberg

**Affiliations:** Klinik für Kardiovaskuläre Chirurgie Universitätsklinikum Düsseldorf peter.feindt@uni-duesseldorf.de

**To the Editor:** The authors presented a therapy for patients with acute cardiopulmonary failure of gaining interest,^1^ since several portable technologies for cardiopulmonary support have become available. However, the different technologies focus on different clinical indications and are suitable for different clinical strategies in providing extracorporeal cardiopulmonary support in emergency care. The aim of this letter is to highlight the key differences of the various systems and to indicate the most promising of these appealing new technologies that have the potential to markedly change the current treatment modalities for acute cardiopulmonary failure.

The clinical value of mechanical support as the optimal treatment for patients with acute circulatory failure currently is under intensive debate. As a result, current guidelines for the use of aortic counter pulsation in acute circulatory failure are questioned.^2^ Advanced systems for mechanical circulatory support such as ECMO (extracorporeal membrane oxygenation) or percutaneously implantable heart-ling transplant systems have been established for emergency support of post-operative patients inside heart centers with circulatory or pulmonary failure.^3^ These systems are ready for use after 30 to 45 minutes, require specialized technical personal for the set up as well as operation, are not qualified for patient transport, but are often designed to provide long-term cardiopulmonary support. Recently, short-term mechanical circulatory support more often is used in cardiogenic shock that can become manifest in a very rapid and often transient loss of circulation.^4^,^5^

**Figure 1:**
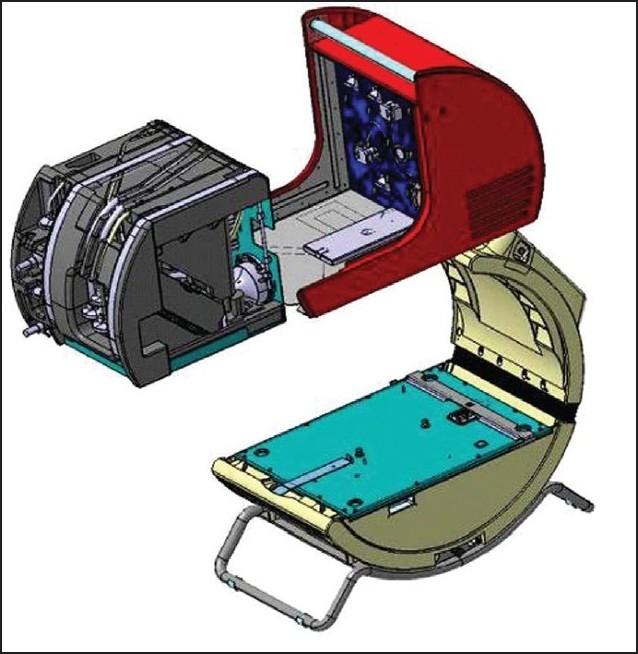
Modular configuration of the LIFEBRIDGE system: the base module (white), the stand alone, portable control module (red) and the patient module (grey, disposable).

With novel techniques of mobile, easy to use heart lung support systems like LIFEBRIDGE B2T (Lifebridge Medizintechnik AG, Ampfing, Germany) it is possible to provide full cardiopulmonary support within several minutes after the patients shows first signs of circulatory crisis.^6^ The major advantage of this modular system (Figure 1) is that it can be applied very quickly and that it can be with access at any time and at any location where circulatory crisis occurs. A recent analysis of patients with acute coronary syndrome and cardiogenic shock by the “Euro Heart Survey” registry did find, that in the vast majority of events (71%) the circulatory crisis occurs in the hospital; the patient often does reach the hospital or already is hospitalized and “crashes” inside the hospital, where advanced health care such as circulatory support can be provided. In case of acute circulatory failure the rapid access of this emerging technology is of key value and it has been shown that it has a high impact on the survival of the treated patients.^7^ The integrated safety features and the semi-automated priming sequence of the system allows trained personal on intensive care units, in the emergency room or cath lab to quickly regain sufficient circulation together with sufficient oxygenation of the patient. Without any delay due to transfer time of equipment and specialized teams from heart centers, the patient quickly can be stabilized by the local team trained on using the mobile LIFEBRIDGE B2T system. De-airing and operation without risk of air emboli is guaranteed by a 7-step air management design that contains different active and passive processes to quickly establish and control extracorporeal cardiopulmonary support.^6^ Within several minutes the system is primed and ready for use.

The cardiac surgeon no longer needs to get to the patient in circulatory crisis, since the rapid extracorporeal cardiopulmonary support can safely be established by a team consisting of an experienced interventional cardiologist or intensive care specialist who implants the femoral cannulas using Seldinger technique and a trained nurse that sets up the system in a semi-automated guided process of priming the system. This procedure serves as a powerful method of avoiding irreversible consequences of cardiogenic shock such as multi-organ failure or disease deterioration. After rapid normalization of arterial blood pressures and oxygenation a reasonable time window for the patient is opened. Extensive diagnostics including CT imaging can be performed under stable hemodynamic and metabolic conditions that lead to optimal organ protection. Once the diagnosis is made and no spontaneous recovery is achieved, the underlying disease can be treated (for example, surgery, high-risk percutaneous coronary intervention), if required under extracorporeal support.

In case of other treatment options patients can safely be transferred to remote hospitals with specialized treatments such as transplant programs or assist programs. Currently, the ECLS (extracorporeal life support or ECMO transports usually are very complex and guidelines for staff and patient safety during transport are not met. However, the Conformité Européenne-marked emergency cardiopulmonary support systems LIFEBRIDGE B2T system is the first and only HLM approved to date for mobile use according European norm EN 1789. With the availability of these novel systems, the clinical use of extracorporeal circulation outside the operating room to treat circulatory or pulmonary failure is expected to increase rapidly in the future, and unaddressed clinical needs in acute intensive care will be met.
